# A Ketogenic Diet in Rodents Elicits Improved Mitochondrial Adaptations in Response to Resistance Exercise Training Compared to an Isocaloric Western Diet

**DOI:** 10.3389/fphys.2016.00533

**Published:** 2016-11-08

**Authors:** Hayden W. Hyatt, Wesley C. Kephart, A. Maleah Holland, Petey Mumford, C. Brooks Mobley, Ryan P. Lowery, Michael D. Roberts, Jacob M. Wilson, Andreas N. Kavazis

**Affiliations:** ^1^School of Kinesiology, Auburn UniversityAuburn, AL, USA; ^2^Department of Health Sciences and Human Performance, University of TampaTampa, FL, USA

**Keywords:** ketogenic diets, mitochondria, antioxidants, exercise, oxidative stress

## Abstract

**Purpose:** Ketogenic diets (KD) can facilitate weight loss, but their effects on skeletal muscle remain equivocal. In this experiment we investigated the effects of two diets on skeletal muscle mitochondrial coupling, mitochondrial complex activity, markers of oxidative stress, and gene expression in sedentary and resistance exercised rats.

**Methods:** Male Sprague-Dawley rats (9–10 weeks of age, 300–325 g) were fed isocaloric amounts of either a KD (17 g/day, 5.2 kcal/g, 20.2% protein, 10.3% CHO, 69.5% fat, *n* = 16) or a Western diet (WD) (20 g/day, 4.5 kcal/g, 15.2% protein, 42.7% CHO, 42.0% fat, *n* = 16) for 6 weeks. During these 6 weeks animals were either sedentary (SED, *n* = 8 per diet group) or voluntarily exercised using resistance-loaded running wheels (EXE, *n* = 8 per diet group). Gastrocnemius was excised and used for mitochondrial isolation and biochemical analyses.

**Results:** In the presence of a complex II substrate, the respiratory control ratio (RCR) of isolated gastrocnemius mitochondria was higher (*p* < 0.05) in animals fed the KD compared to animals fed the WD. Complex I and IV enzyme activity was higher (*p* < 0.05) in EXE animals regardless of diet. SOD2 protein levels and GLUT4 and PGC1α mRNA expression were higher (*p* < 0.05) in EXE animals regardless of diet.

**Conclusion:** Our data indicate that skeletal muscle mitochondrial coupling of complex II substrates is more efficient in chronically resistance trained rodents fed a KD. These findings may provide merit for further investigation, perhaps on humans.

## Introduction

Low carbohydrate ketogenic diets (KD) that induce nutritional ketosis have been successfully utilized in the treatment of multiple health disorders ranging from obesity to neurodegenerative diseases such as Parkinson's disease (Gasior et al., 2006; Wylie-Rosett et al., 2013; Paoli, 2014). Although many of these disorders are also associated with mitochondrial dysfunction (Kelley et al., 2002; Ritov et al., 2005; Lin and Beal, 2006), the majority of previous research regarding mitochondrial function and a KD has been centered around the effects of a KD on neuronal tissue in the brain. While mitochondria are a diverse organelle capable of responding to a multitude of metabolic stressors such as aerobic and/or resistance exercise and caloric restriction (Gredilla et al., 2001; Menshikova et al., 2006; Salvadego et al., 2013; Porter et al., 2015), differing metabolic profiles exist between the brain and skeletal muscle due to the blood brain barrier (Pardridge, 1983). Currently the effects of a KD on skeletal muscle mitochondria are not well understood.

While dietary effects on mitochondrial functioning can be difficult to delineate, previous research has elucidated possible mechanisms that implicate a modulatory role for KD capable of impacting mitochondrial function. KD has been demonstrated to increase AMP-activated protein kinase (AMPK) signaling in skeletal muscle and may serve as an important mechanistic drive to provide mitochondrial alterations (Kennedy et al., 2007). AMPK acts to phosphorylate proliferator-activated receptor γ coactivator 1α (PGC-1α), which is a key transcription factor involved in mitochondrial biogenesis (Wu et al., 1999). Additionally, ketogenic diets increase the circulation of free fatty acids which are capable of activating peroxisome proliferator-activated receptor α (PPARα), a nuclear transcription factor responsible for upregulating genes involved in lipid oxidation in the mitochondria (Leone et al., 1999; Badman et al., 2009). Furthermore, the ketogenic diet is also demonstrated to affect systems involved in mediating oxidative stress (Milder et al., 2010).

Observation of the physiological alterations imposed by a low carbohydrate ketogenic diet on mitochondrial function is imperative to understand the potential benefits and consequences a KD provokes in skeletal muscle. Considering that exercise is also a viable treatment for both weight loss and certain types of neurodegenerative diseases, observing the physiological adaptations to a combination of exercise and KD can provide useful insight for what could potentially be translated into effective clinical treatments for these conditions. We observed the effects of 6 weeks of Western diet (WD) and KD feeding in animals that were either sedentary or voluntary resistance-wheel trained; notably, all animals were provided isocaloric amounts of each diet. Specifically, we hypothesized that greater adaptations of mitochondrial function would occur in animals fed a KD in response to resistance-wheel exercise.

## Materials and methods

### Animals

All experimental procedures were approved by Auburn University's Institutional Animal Care and Use Committee (IACUC, protocol # 2015-2612). Male Sprague-Dawley rats ~9–10 weeks of age (~300–325 g) were purchased (Harlan Laboratories, Indianapolis, IN, USA) and allowed to acclimate in the animal housing facility for 1 week prior to experimentation. During acclimation, animals were provided standard rodent chow (24% protein, 58% CHO, 18% fat; 114 Teklad Global #2018 Diet, Harlan Laboratories) and water *ad libitum* in a maintained ambient temperature and constant 12 h light: 12 h dark cycle. The animals for this study were the same animals previously published by Roberts et al. (2016) who reported that KD and WD feeding promoted similar increases in muscle mass with the employed resistance-loaded running wheel regimen.

For a 6-week period after acclimation, animals were provided isocaloric amounts of one of two diets:

1. 16 animals were provided with 17 g/day of a putative commercially designed LCKD (Harlan Tekland diet #10787) that was designed to induce nutritional ketosis. The diet specifications were as follows: 5.2 kcal/g, 20.2% protein, 10.3% CHO, 69.5% fat. Medium chain triglycerides, flaxseed oil and canola oil were prominent fat sources. Casein was the sole protein source and maltodextrin was the sole CHO source.
2. 16 animals were provided with 20 g/day of a WD (Harlan Tekland diet #88137). The diet specifications were as follows: 4.5 kcal/g, 15.2% protein, 42.7% CHO, 42.0% fat. Anhydrous milkfat was the prominent fat source. Casein was the sole protein source and sucrose and corn starch were the main CHO sources. Of note, 34% of the CHOs were from sucrose which makes this a high-fat/high-glycemic chow.

A ¼ tablespoon of artificial sweetener (Splenda) was added to the KD diets for only the first week of feeding in order to encourage food consumption. Animals from each diet described above were assigned to either a chronically sedentary condition (WD SED, *n* = 8, KD SED, = 8) whereby they were doubly housed in standard rat cages for social enrichment per the IACUC's recommendation, or they were enrolled in a chronic rat resistance training model (WD EX, *n* = 8; KD EX, *n* = 8) whereby they were individually housed in a cage with a resistance-loaded voluntary running wheel (Lafayette Instrument Company, Lafayette, IN, USA). During the acclimation phase EX animals performed voluntary free-wheel running on *ad libitum* standard chow diets to allow them adapt to running cages (days -7 to -1). During days 1–8, the diets began and the wheel remained unloaded so that the rats could adapt to their respective diets. A wheel resistance of 20–25% body mass was then applied during days 8–15, a resistance of 40% body mass was applied during days 16–24, a resistance of 60% body mass was applied during days 25–32, and a resistance of 40% body mass was applied during days 33–42. The wheel resistance was dropped from 60 to 40% during the last phase given that several rats were not voluntarily running at the 60% resistance workload. All groups were provided water *ad libitum* during the 6-week intervention.

On the day of euthanasia, food was deprived in both groups for ~6 h but animals were provided water *ad libitum*. Additionally, animals in the EX group had their running wheels locked 24 h prior to the day of euthanasia. Rats were placed under isoflurane anesthesia then euthanized under CO_2_ gas in a 2 L induction chamber (VetEquip, Inc., Pleasanton, CA, USA).

### Tissue preparation

About 800 mg from the right gastrocnemius muscle was immediately used for mitochondria isolation as described below. Approximately 50 mg of the left gastrocnemius was placed in 10 volumes of ice cold cell lysis buffer for Western blotting, and another 50 mg of the left gastrocnemius was placed in 10 volumes of Ribozol (Ameresco) for mRNA analyses (both described below).

### Mitochondrial isolation and respiration

Differential centrifugation was used to isolate gastrocnemius mitochondria as described previously (Kavazis et al., 2010). Mitochondrial oxygen consumption was measured as described by Messer et al. (Messer et al., 2004) in a respiration chamber maintained at 37°C (Hansatech Instruments). Isolated mitochondria were incubated with 1 mL of respiration buffer containing (in mM) 100 KCl, 5 KH_2_PO_4_, 1 EGTA, 50 MOPS, 10 MgCl_2_, and 0.2% BSA at 37°C in a water-jacketed respiratory chamber with continuous stirring. Flux through *complex I* was measured using 2 mM pyruvate and 2 mM malate, whereas flux through *complex II* was measured using 5 mM succinate. Rotenone (5 μM) was added to prevent electron backflow to *complex I* in the succinate-driven experiments. The maximal respiration (*state 3*), defined as the rate of respiration in the presence of ADP, was initiated by adding 0.25 mM ADP to the respiration chamber containing mitochondria and respiratory substrates. *State 4* respiration was recorded following the phosphorylation of ADP. The respiratory control ratio (RCR) was calculated by dividing *state 3* by *state 4* respiration.

### Western blotting

Approximately 50 mg of gastrocnemius muscle was obtained using standard dissection techniques and immediately placed in 500 μL of ice-cold cell lysis buffer (Cell Signaling; 20 mM 257 Tris-HCl (pH 7.5), 150 mM NaCl, 1 mM Na-EDTA, 1 mM EGTA, 1% Triton, 20 mM sodium pyrophosphate, 25 mM sodium fluoride, 1 mM β-glycerophosphate, 1 mM Na_3_VO_4_, 1 μg/mL leupeptin). Samples were then homogenized via micropestle manipulation, and insoluble proteins from homogenates were removed with centrifugation at 500 × g for 5 min. Homogenates were then stored at −80°C. Protein determination on cell lysis homogenates was performed using a BCA Protein Assay Kit (Thermo Scientific, Waltham, MA, USA). Homogenates were prepared for Western blotting using 4x Laemmli buffer at 2 μg/μL. Equal amount of proteins were separated by polyacrylamide gel electrophoresis via 12% polyacrylamide gels containing 0.1% sodium dodecyl sulfate for ~2 h at 120 V (C.B.S. Scientific Company, San Diego, CA). After electrophoresis, the proteins were transferred to polyvinylidene difluoride membranes (Amresco) via the C.B.S. Scientific Company system for 2 h at 200 mA. Nonspecific sites were blocked for 1 h at room temperature in PBS solution containing 0.05% Tween and 5% nonfat milk. Membranes were then incubated for 1 h with primary antibodies directed against the proteins of interest. The primary antibodies used were superoxide dismutase 2 (SOD2; # GTX116093; GeneTex, Irvine, CA), catalase (# GTX110704; GeneTex), and 4-hydroxynonenal-conjugated proteins (4-HNE, # ab46545; Abcam, Cambridge, MA). Following incubation with primary antibodies, membranes were washed extensively with PBS–Tween and then incubated with secondary antibodies. Membranes were then developed using an enhanced chemiluminescent reagent (Amersham, Pittsburgh, PA), and band densitometry was performed through the use of a UVP Imager and associated densitometry software (UVP, LLC, Upland, CA). Ponceau staining was used as the normalizing control.

### RT-PCR for gastrocnemius mRNA expression

About 20-40 mg of muscle was pre-weighed and placed in 10 volumes Ribozol (Ameresco) in a 1.7 mL microcentrifuge tube (Thermo Scientific). Thereafter, muscle was homogenized using a tight-fitting pestle. Phase separation (for RNA and protein isolation) was achieved using a modification to the manufacturer's instructions; specifically, 1-bromo-3-chloropropane was used in place of chloroform. Following RNA precipitation, pellets were re-suspended in 40 μL of RNase-free water, concentrations were determined in duplicate at an absorbance of 260 nm using a NanoDrop Lite (Thermo Scientific), and concentrations were normalized to muscle mass.

After total RNA determination described above, 2 μg of gastrocnemius RNA was reverse transcribed into cDNA for real-time PCR (RT-PCR) analyses using a commercial qScript™ cDNA SuperMix (Quanta Biosciences, Gaithersburg, MD, USA). RT-PCR was performed using gene-specific primers and SYBR-green-based methods in a RT-PCR thermal cycler (Bio-Rad Laboratories, Hercules, CA, USA). Primers were designed using primer designer software (Primer3Plus, Cambridge, MA, USA). The forward and reverse primer sequences are as follows: glucose transporter type 4 (GLUT4) forward primer; 5′-GCTTCTGTTGCCCTTCTGTC-3′ and reverse primer 5′-TGGACGCTCTCTTTCCAACT-3′; peroxisome proliferator-activated receptor gamma coactivator 1-alpha (PGC-1α) forward primer 5′-ATGTGTCGCCTTCTTGCTCT-3′ and reverse primer 5′-ATCTACTGCCTGGGGACCTT-3′; carnitine palmitoyltransferase 1B (CPT1B) forward primer 5′-GCAAACTGGACCGAGAAGAG-3′ and reverse primer 5′-CCTTGAAGAAGCGACCTTTG-3′; pyruvate dehydrogenase kinase, isozyme 4 (PDK4) forward primer 5′- AAAGTGGGTCTACGGCAGTG -3′ and reverse primer 5′- TGCGGAAACAAGAGTCCACA -3′; histone deacetylase 1 (HDAC1) forward primer 5′- GAGCGGTGATGAGGATGAGG -3′ and reverse primer 5′- CACAGGCAATGCGTTTGTCA -3′. HDAC1 expression was used as the reference gene. Relative quantification of gene expression was performed using the 2ΔΔCT method whereby ΔCT [CT(reference gene)−CT(gene of interest)].

### Mitochondrial complex activity

Enzymatic assays to determine electron transport chain complex activity was performed as described previously (Kavazis et al., 2009). Mitochondria isolated from the gastrocnemius were subjected to three cycles of freezing and thawing to lyse membranes before analysis. Complex I (NADH dehydrogenase) enzyme activity was measured as a function of the decrease in absorbance from NADH oxidation by decylubiquinone before and after the addition of rotenone. Complex II (succinate dehydrogenase) activity was measured as a function of the decrease in absorbance from 2,6-dichloroindophenol reduction. Complex III (ubiquinol cytochrome coxidoreductase) activity was determined as a function of the increase in absorbance from cytochrome c reduction. Complex IV (cytochrome c oxidoreductase) activity was determined as a function of the decrease in absorbance from cytochrome c oxidation. Specificity of complex IV activity was determined by monitoring changes in absorbance in the presence of KCN. Citrate synthase was measured as a function of the increase in absorbance from 5,5′-dithiobis-2-nitrobenzoic acid reduction. Enzyme activities are expressed as a ratio to citrate synthase to compensate for mitochondrial enrichment in the cell samples.

### Statistical analysis

All data are presented as means ± standard deviation. A two-way (diet^*^exercise) ANOVAs were used and statistical significance was set at *p* < 0.05.

## Results

### Mitochondrial oxidative phosphorylation

**Figure 1:**
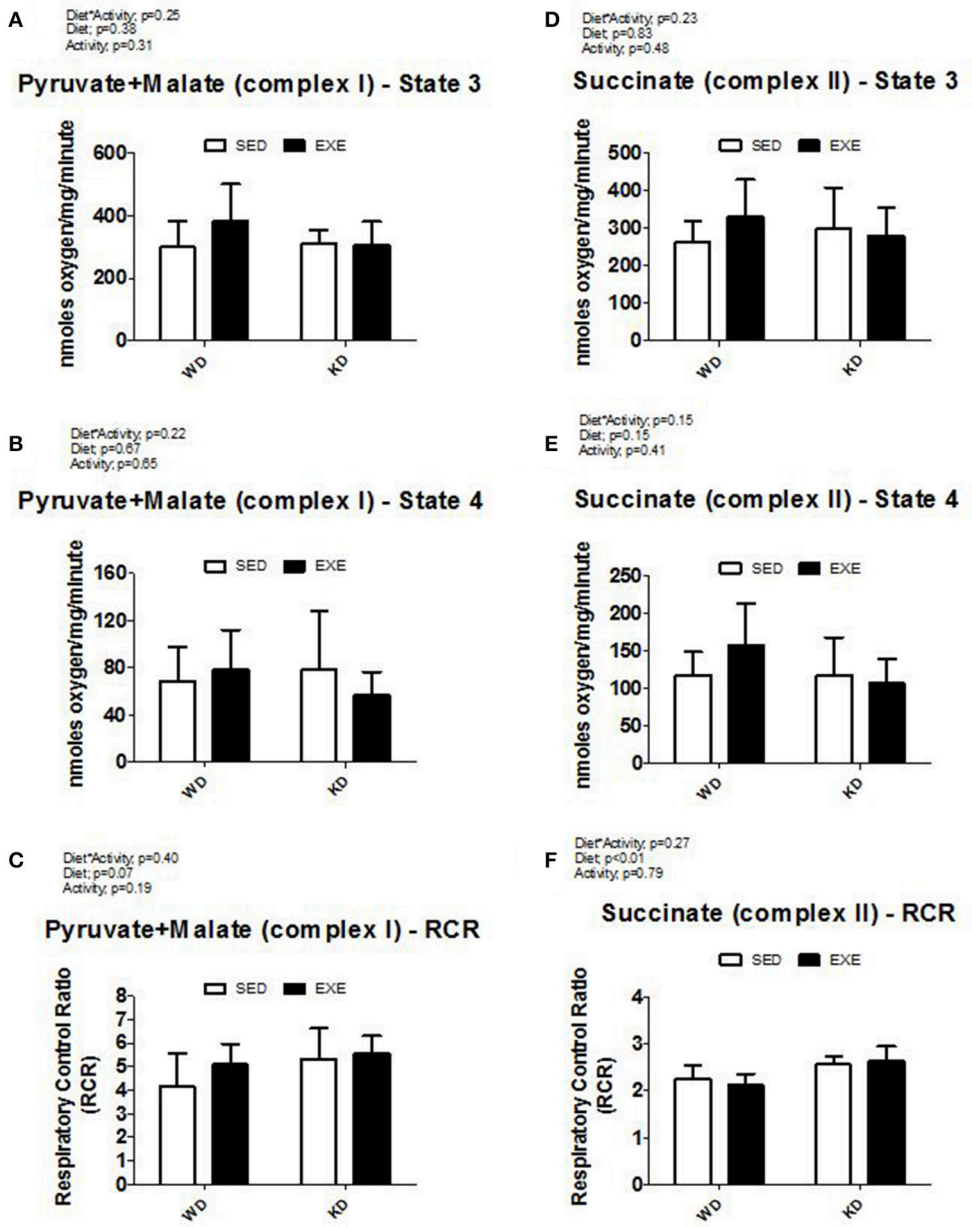
**Isolated mitochondria respiration. (A)** State 3 and **(B)** State 4 respiration with pyruvate and malate (complex I), **(C)** respiratory control ratio (RCR) with pyruvate and malate (complex I), **(D)** State 3 and **(E)** State 4 respiration with succinate (complex II), and **(F)** respiratory control ratio (RCR) with pyruvate and malate (complex II).

Isolated mitochondria respiration was obtained in the presence of complex I (pyruvate+malate) or complex II (succinate) substrates (Figure 1). KD-fed animals presented a higher (diet effect *p* < 0.01) mitochondria complex II respiratory control ratio (RCR) compared to WD-fed animals, but no differences were detected when complex I was used.

### Mitochondrial complex activity and skeletal muscle citrate synthase

**Figure 2:**
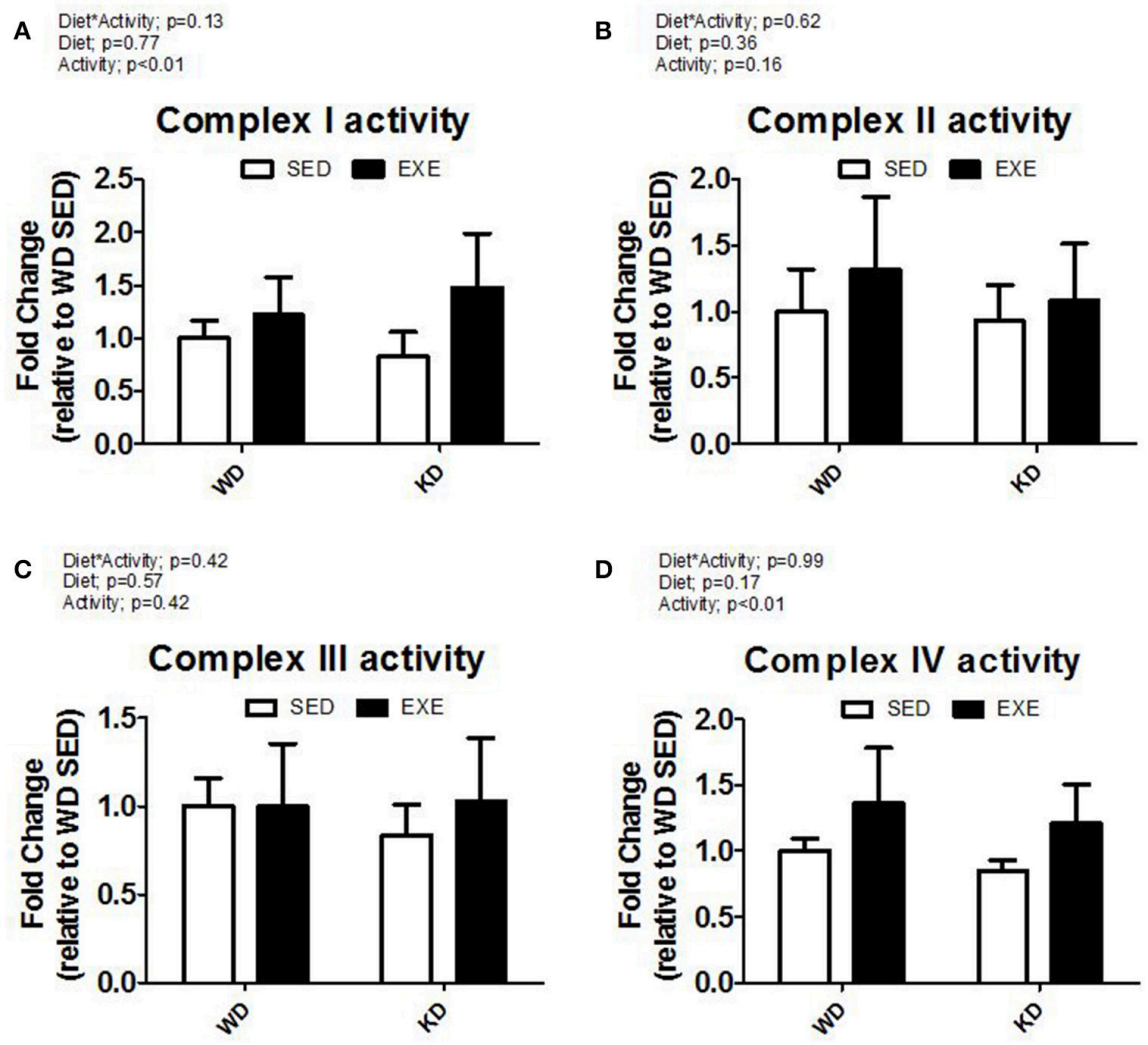
**Isolated mitochondria complex activity. (A)** Complex I, **(B)** complex II, **(C)** complex III, and **(D)** complex IV activity.

**Figure 3:**
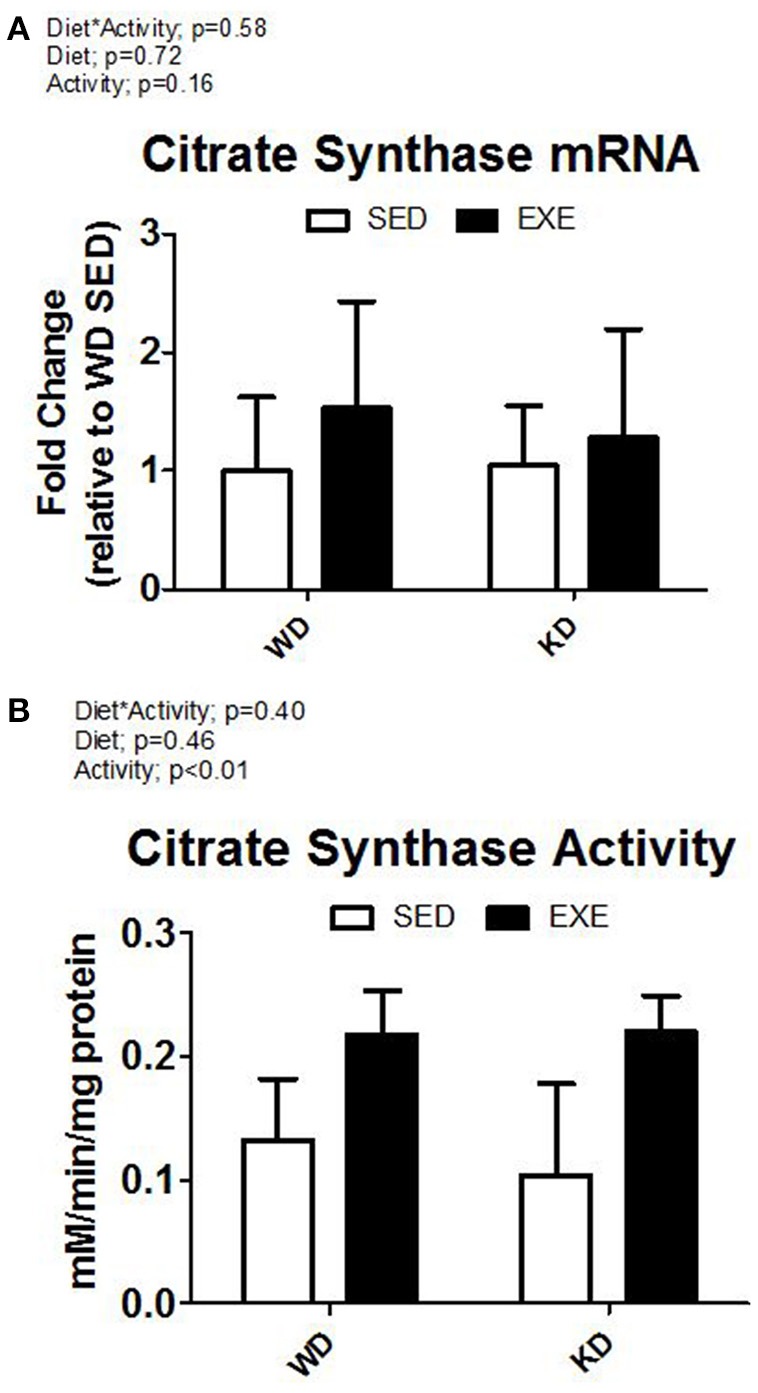
**Citrate synthase mRNA and enzyme activity. (A)** mRNA expression of citrate synthase, and **(B)** citrate synthase activity from whole muscle homogenate.

The enzymatic activity of complex I, II, III, and IV were measured in isolated mitochondria (Figure 2). A main effect of activity (*p* < 0.01) was found for complex I and complex IV. Specifically, the enzymatic activity of complex I and complex IV was about 50 and 40% higher in the EXE animals. No differences were detected for complex II or III. Citrate synthase mRNA expression was higher in EXE animals, albeit this did not reach statistical significance (Figure 3). However, the citrate synthase enzyme activity assay did show a significant (*p* < 0.01) effect of activity (i.e., EXE animals had about 40% higher activity).

### Antioxidants and markers of oxidative damage

**Figure 4:**
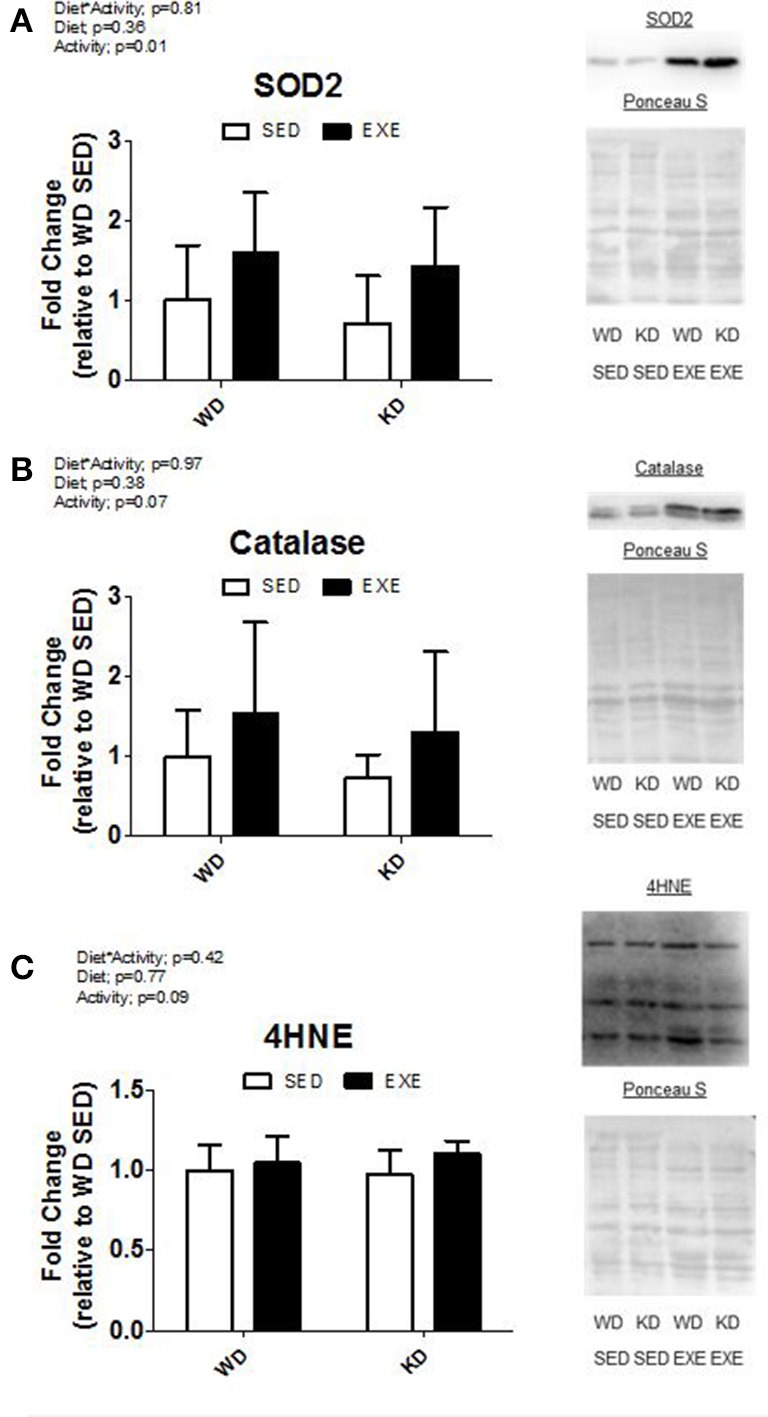
**Antioxidant protein levels and marker of oxidative damage**. Protein expression of **(A)** superoxide dismutase 2 (SOD2), **(B)** catalase, and **(C)** 4-hydroxynonenal-conjugated proteins (4-HNE). Representative Western blot images are shown to the right of the graph.

The protein levels of two antioxidants (SOD2 and catalase) and a marker of oxidative damage (4-HNE) were measured (Figure 4). A main effect of activity (*p* = 0.01) was present in SOD2. Specifically, the SOD2 protein levels were about 75% higher in the EXE animals. Exercise increased catalase protein levels, but no significance was reached (*p* = 0.07). There were no differences in 4-HNE.

### mRNA expression of mitochondrial and metabolic markers in skeletal muscle

**Figure 5:**
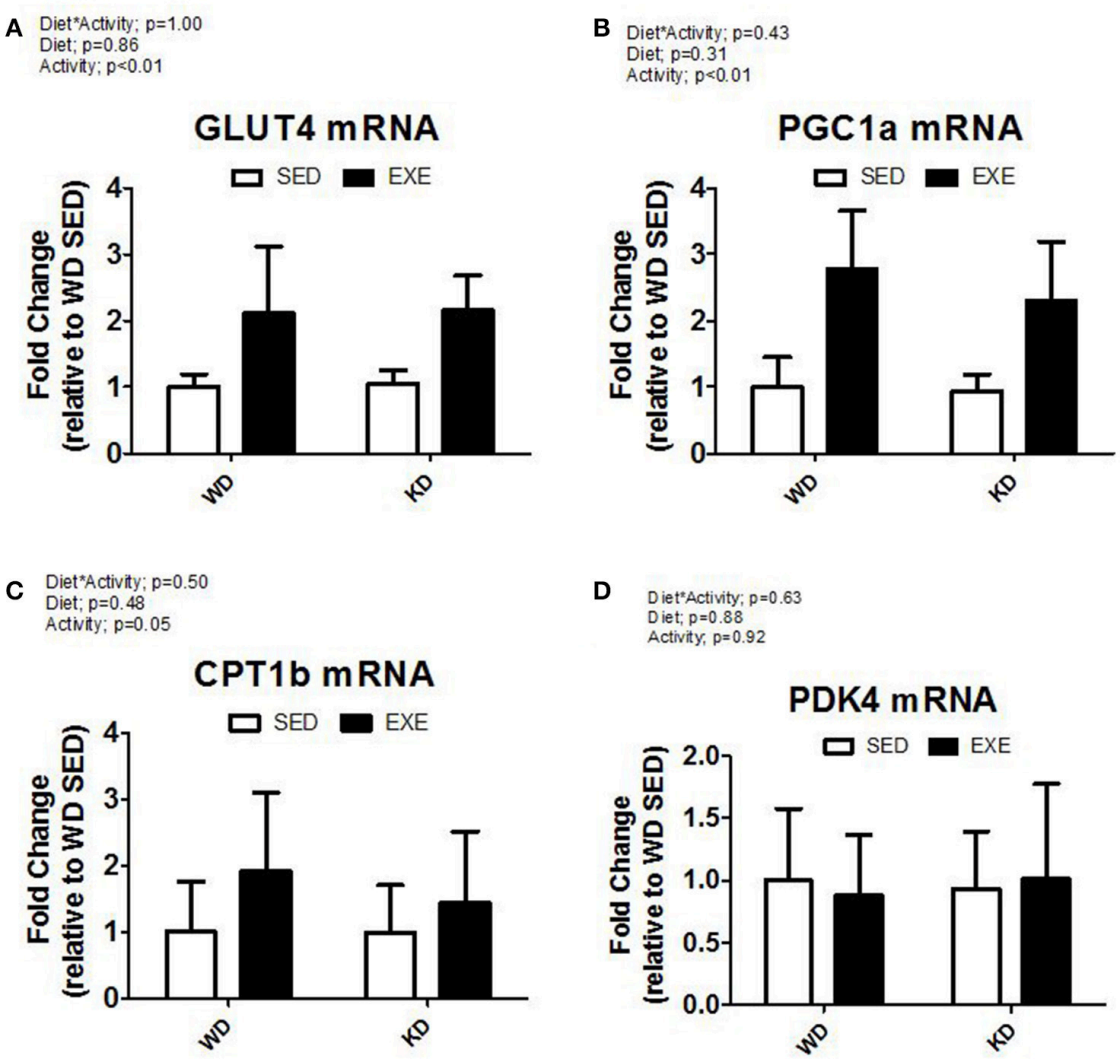
**mRNA expression of key metabolic genes. (A)** Glucose transporter type 4 (Glut4), **(B)** peroxisome proliferator-activated receptor gamma, coactivator 1 alpha (PGC1α), **(C)** Carnitine Palmitoyltransferase 1B (CPT1B), and **(D)** Pyruvate Dehydrogenase Kinase, Isozyme 4 (PDK4).

The mRNA expression of select genes was also measured (Figure 5). A main effect of activity (*p* < 0.01) was present in GLUT4 mRNA expression and PGC1α with EXE animals having about a 2-fold and 2.5-fold increase, respectively. CPT1b mRNA expression increased in EXE animals, but no significance was detected (*p* = 0.05). No statistically significant differences occurred in mRNA expression for PDK4.

## Discussion

Studies observing the effects of a KD on mitochondrial function have primarily focused on neuronal tissue in the brain. However, mitochondria located in the brain experience a vastly different metabolic profile than skeletal muscle due to the blood-brain barrier (Pardridge, 1983), thus a KD likely emanates differing signaling events between skeletal muscle and brain mitochondria. These experiments provide novel information on gastrocnemius mitochondrial function and adaptation to exercise in WD and KD animals. Our findings show that mitochondrial adaptations occur after exercise training in both WD animals and KD animals. Moreover, we show for the first time that mitochondrial adaptations occurred in response to the KD and resulted in higher RCR of complex II in the mitochondria from skeletal muscle. Furthermore, animals exposed to resistance wheel exercise training had increases in mitochondrial complex activity, mRNA expression of genes involved in metabolism, and endogenous antioxidant protein expression. Previous observation on the hypertrophic response of these animals by Roberts et al. demonstrated increased markers of ketogenesis indicating that KD-fed animals expressed “mild” ketosis (Roberts et al., 2016).

### KD-fed animals have higher coupling at complex II

We report that isolated mitochondria from KD-fed animals have higher RCR while respiring using succinate (i.e., a complex II substrate). To our knowledge, the effects of a KD on mitochondrial respiration have not been observed in skeletal muscle, although studies on other tissue types and derivatives of high fat non-ketogenic diets may be applicable to the interpretation of our findings. Recent findings have implicated that high fat diets lead to increased respiration in skeletal muscle mitochondria. Jorgenson et al. reported that a 1 year high fat diet fed to rats resulted in 30 to 50% increases in respiration compared to a standard chow control group (Jorgensen et al., 2015). Iossa et al. also reported that a more acute 15-day high fat diet resulted in increases of state 3 and state 4 respiration with complex I substrates but no changes in complex II RCR from mitochondria isolated from skeletal muscle (Iossa et al., 2002). Findings of increased respiration may be explained in part by evidence that high fat diets increase uncoupling proteins (i.e., UCP3) in skeletal muscle (Turner et al., 2007). While the differing experimental protocols and length of the diet complicate cross comparison of the findings, the contrast between reports of uncoupled mitochondria from high fat diets and our current findings may be best explained by the fat sources utilized. The aforementioned studies contained fat sources derived primarily from animal fats that are high in long-chain triglycerides (i.e., lard and butter). The putative KD utilized in the current study contained fat sources primarily from medium chain triglycerides (i.e., MCTs, canola oil, and flaxseed oil). Interestingly, it has been reported that long-chain triglycerides, but not medium-chain triglycerides, can result in uncoupling of mitochondrial respiration along with increased uncoupling protein expression (Murray et al., 2011). This observation would explain the increased uncoupling protein expression and respiration rates described in previous studies, while our current findings may be explained by long-chain triglycerides in the WD-fed animals causing an uncoupling stimulus in complex II and lack of this stimulus in KD-fed animals. It should be noted though, that complex II has been indicated to serve an important role in response to the ketogenic diet. Subcutaneous injections of the ketone body, d-β-hydroxybutyrate, has been reported to alleviate symptoms of Parkinson's disease in mouse brain due to increased functioning of complex II (Tieu et al., 2003). Thus, our findings of increased coupling of complex II in KD fed animals compared to WD fed animals may be due to currently unknown biochemical interactions between the signaling pathways activated by ketone bodies and/or medium-chain triglycerides that affect mitochondrial functioning.

### Improvements of mitochondrial protein activity with resistance-wheel training

Resistance-wheel training resulted in increased activity of complex I and IV in isolated mitochondria. While studies have shown increased complex activity in whole muscle homogenates and isolated muscle fibers in response to exercise training (Holloszy, 1967; Wibom et al., 1992), observations on mitochondrial enzyme activity in whole muscle homogenate is subject to a multitude of factors and can reflect increases in the relative amount of the enzymes or alterations in enzyme functionality. As such, our measurements of enzyme activity in isolated mitochondria likely indicate that the complex enzymatic function was increased in response to resistance-wheel training, perhaps due to post-translational modifications. Although the interaction effect was not significant, animals fed the WD had about a 20 and 35% increase in complex I and complex IV activities following exercise. However, animals fed the KD had about 80 and 45% increase in complex I and complex IV activities following exercise. It would appear that the KD is more effective in conferring mitochondrial adaptations in response to exercise training compared to WD. This observation could be a result of increased AMPK activation, an important signaling event in mitochondrial biogenesis and function (Kennedy et al., 2007), induced by KD. However, previous report on these animals observed no differences in p-AMPK (Roberts et al., 2016). This observation was attributed to adaptation to the ketogenic diet and a resetting of AMPK signaling, thus the differences in significance from our current findings may be due to adaptations earlier in training while adaptation to the KD was taking place (Roberts et al., 2016).

### Increase in markers of metabolism and mitochondrial biogenesis in response to resistance-wheel training

Resistance wheel trained animals had higher citrate synthase activity in whole muscle homogenate in both diets and was accompanied by a non-significant (*p* = 0.16) increase in citrate synthase mRNA. Citrate synthase activity can be utilized as a predictor of mitochondrial content (Jackman and Willis, 1996). Our findings of similar increases in citrate synthase activity between both diets suggests that mitochondrial biogenesis was similar in response to the exercise training. Furthermore, the finding that citrate synthase activity was not different between groups supports our conclusion that the reported increase in complex activity represents an increase in function rather than alterations in enzyme concentration. Interestingly, previous research involving high-fat diets have provided conflicting data on the impact of mitochondrial content. Sparks et al. showed that a high-fat diet downregulates genes involved in oxidative phosphorylation in skeletal muscle (Sparks et al., 2005), while Hancock et al. reported that a high-fat diet actually increased markers of mitochondrial content in skeletal muscle (Hancock et al., 2008). We report that PGC-1α mRNA expression, a potent stimulator of mitochondrial biogenesis and fatty acid oxidation (Wu et al., 1999; Vega et al., 2000), was increased with 6 weeks of resistance wheel training regardless of diet. Thus it would appear that if increases in mitochondrial content in response to high fat feedings does occur, increases in mitochondrial content is normalized with exercise training and perhaps KD feeding effectuates a concurrent increase in mitochondrial function.

### Oxidative stress

An imbalance between antioxidants and reactive oxygen species can lead to oxidative damage. Reports on markers of oxidative stress in animals that are fed a KD for 3 weeks have been reported to enhance the antioxidant capacity in the rat hippocampus (Jarrett et al., 2008). One mechanism that increases antioxidant capacity in response to a KD is activation of NF E2-related factor 2 (Nrf2), which upregulates transcription of genes involved in protection against oxidative damage. 4-HNE is a possible activator of Nrf2 and an acute KD for 3 days has been demonstrated to increase hippocampal 4-HNE (Milder et al., 2010). Our current results demonstrate no significant differences in 4HNE in whole muscle homogenate. Thus, increased expression of 4-HNE may be limited to acute KD feeding or alternate signaling in hippocampal tissue. SOD2 protein expression was increased following resistance wheel training regardless of diet.

## Conclusions

Ketogenic diets have demonstrated promising health benefits in human populations in terms of weight loss and neurodegenerative diseases (Gasior et al., 2006; Paoli, 2014), however the effects of these diets on mitochondrial function in skeletal muscle has not been well observed. We report that a putative KD resulted in increased mitochondrial RCR when a complex II substrate (i.e., succinate) was used. Six weeks of resistance wheel training resulted in higher enzymatic activity at complex I and IV from isolated mitochondria, higher citrate synthase activity in whole muscle homogenate, higher mRNA expression of PGC-1a and GLUT4, and higher SOD2 protein expression. Our findings of increased RCR at complex II in KD animals may implicate the importance of fat source types on mitochondrial function. Furthermore, our findings also provide merit to obese individuals utilizing a KD in order to lose weight, as mitochondrial function is disrupted in patients with type two diabetes and obesity (Kelley et al., 2002; Ritov et al., 2005). Thus the combined weight loss and increased mitochondrial coupling effects of KD could potentiate the transition of individuals toward a healthier phenotype. These results are important, but we should note that data were obtained at one time point and the animals consumed macronutrients derived from different sources. Therefore, future research is needed to determine if similar effects of a KD on mitochondrial function is apparent in humans and to determine the mechanisms involved in these adaptations.

## Author contributions

AK, MR, and JW: Study concept and design; HH, WK, AH, PM, CM, and RL, MR, JW, and AK: Data analyses and interpretation; HH, AK, and MR: Drafting and critical revision of the article for important intellectual content; WK, AH, PM, CM, and RL: Precious help with editing the manuscript at different stages; AK, MR, and JW: Obtained funding; AK, MR: Study supervision.

### Conflict of interest statement

The authors declare that the research was conducted in the absence of any commercial or financial relationships that could be construed as a potential conflict of interest.
